# 19-year-old Woman with Intermittent Weakness

**DOI:** 10.5811/cpcem.2021.4.52011

**Published:** 2021-07-27

**Authors:** Garrett A. Cavaliere, Neeraja Murali, Laura J. Bontempo, Zachary D.W. Dezman

**Affiliations:** * University of Maryland Medical Center, Department of Emergency Medicine, Baltimore, Maryland; † University of Maryland School of Medicine, Department of Emergency Medicine, Baltimore, Maryland; ‡ University of Maryland School of Medicine, Department of Epidemiology and Public Health, Baltimore, Maryland

**Keywords:** Clinicopathological cases, neurology, renal tubular acidosis

## Abstract

**Introduction:**

Systemic weakness is a common chief complaint of patients presenting to the emergency department (ED). A well thought out approach to the assessment and workup of these patients is key to diagnostic accuracy and definitive therapy.

**Case Presentation:**

In this case, a 19-year-old female presented to the ED with generalized weakness and near syncope. She had global weakness in her extremities and multiple electrolyte abnormalities.

**Discussion:**

This case takes the reader through the differential diagnosis and evaluation of a patient with weakness and profound electrolyte derangements. It includes a discussion of the diagnostic studies and calculations that ultimately led to the patient’s diagnosis.

## CASE PRESENTATION (DR. CAVALIERE)

A 19-year-old female was brought to the emergency department (ED) by emergency medical services (EMS) with complaints of generalized weakness, an inability to move her extremities, and near syncope. The patient stated that she began feeling generalized weakness that morning, which she initially attributed to her “sleeping position.” Over the day the weakness worsened, culminating in difficulty or inability to move her extremities and a near syncopal episode. The patient stated she had attempted to stand up from a seated position when she “felt like [she] was going to pass out.” The patient called 911 for assistance. On further discussion, the patient revealed she had experienced one similar episode of weakness earlier in the year, but this had resolved spontaneously and was not as severe. She does not have a primary care physician and she had never sought care for this complaint. The patient said she noticed generalized abdominal pain, nausea, and constipation, associated with each of these episodes of weakness and lightheadedness. She denied any recent illnesses. She stated she treats her bipolar disorder with daily cannabis and consumes alcohol daily as well.

The patient had a history of anxiety, depression, migraines, and normocytic anemia. Surgical history included an adenoidectomy and tonsillectomy as a child. She had no pertinent family history. Her social history included daily alcohol use, drinking a total of 1.75 liters of vodka over a two-week period. She started smoking when she was 16 years old, smoking a pack per day, but quit a year prior to presentation. The patient smoked cannabis daily. Her only medication was ferrous sulfate 325 milligrams (mg) daily. She had no known drug or environmental allergies.

On physical exam, the patient was alert and in no acute distress but appeared tired. She was able to stand unassisted. At the time of triage, she was afebrile (36.6^o^ Celsius), her heart rate was 40 beats per minute, she was breathing 20 times per minute, her blood pressure was 115/90 millimeters of mercury, and she had an oxygen saturation of 98% on room air. She weighed 77.3 kilograms and was 1.65 meters tall (body mass index = 28.3). She was well developed, well nourished and speaking in complete sentences without accessory muscle use. She was oriented as to person, place and time. She was without sensory deficits and had normal muscle tone. Her strength was 4/5 with elbow flexion and extension, hand grip, knee flexion and extension, and ankle dorsi- and plantar-flexion bilaterally. Deep tendon reflexes were 2+ for the bilateral brachioradialis and patellar reflexes. No clonus could be provoked.

She did not have any cranial nerve (II–XII) defects, and she had a normal gait and station. She had normal range of motion of all four extremities, and she did not have any edema. Her lower extremity compartments were soft in both the thighs and the lower legs bilaterally. She exhibited tenderness around her bilateral shoulders and shins. Her head was normocephalic and without signs of injury. Her oropharynx was clear and moist, and her pupils were equal, round, and reactive to light. Her conjunctiva and extraocular motions were normal. Her neck was supple and had a full range of motion, without jugular venous distention or adenopathy.

On cardiovascular exam the patient was bradycardic with a regular rhythm, and she had a normal S1/S2 without gallops, friction rubs, or murmurs. On auscultation her breath sounds were clear without wheezes, rales or rhonchi. Her abdomen was non-distended, soft and non-tender throughout with normal bowel sounds. Her skin was warm and dry, and her capillary refill was less than two seconds. She did not have any rashes. Her mood, affect, and behavior were normal. The patient’s electrocardiogram (ECG) is shown (Image). The results of the patient’s initial laboratory evaluation are shown in Table 1. A test was ordered, and a diagnosis was made.

## CASE DISCUSSION (DR. MURALI)

This case involved a young woman with episodic weakness. She reported near syncope, transient extremity paralysis, and generalized weakness. She reported associated nausea, abdominal pain, and constipation. She also reported regular substance use in the form of marijuana and alcohol. Her review of systems was otherwise unremarkable, and notably it was negative for recent illness or gastrointestinal (GI) distress outside of this episode.

With this in mind, I began to formulate a differential diagnosis. Episodic weakness, particularly extremity paralysis, suggests metabolic and electrolyte derangements such as hypokalemic periodic paralysis. Weakness may also suggest a primary neurologic condition, including Guillain-Barré syndrome, multiple sclerosis, and other demyelinating disorders. The patient’s near syncope may be due to orthostatic hypotension or neurocardiogenic causes. Her GI symptoms could be due to a broad array of abdominal conditions, but her substance use suggests these symptoms may be related to an ingestion. The patient’s bradycardia could be due to disseminated Lyme disease, myocarditis, or other etiologies of heart block. More information is required.

I used the information provided by her physical exam to further refine my differential diagnosis. Her physical exam was notable for a tired-appearing female with bradycardia. Pertinent negative findings included that the compartments of the legs were noted to be soft, clinically excluding a compartment syndrome. Additionally, the patient had no focal neurologic deficits based on the documented neurologic exam. Several findings, including cerebellar signs were not documented, but the patient was noted to have normal gait and station. The mention of normal compartments and the normal neurologic exam suggests that a neurologic cause is unlikely. Further, the case did not provide any imaging studies – notably, there was no neuroimaging included. The patient’s ECG showed a sinus bradycardia with sinus arrhythmia, short QTc with T-wave inversions in aVR and V1, and U waves, but it did not show a heart block.

I then reviewed the patient’s laboratory findings. She was noted to have a mild anemia, elevated creatine kinase with myoglobinuria, hematuria, proteinuria, and urinary findings consistent with a urinary tract infection. Additionally, she has multiple electrolyte derangements, including hypokalemia, hyperchloremia with acidosis, hypermagnesemia and hypophosphatemia. She had an elevated creatinine and a mild transaminitis. These laboratory findings suggest her symptoms are due to a metabolic derangement.

This patient had a non-anion gap metabolic acidosis. The differential diagnosis for non-anion gap metabolic acidosis includes diarrhea, intestinal fistulae, renal tubular acidosis (RTA), ureteroileostomy, ureterosigmoidostomy, toluene use, ketoacidosis, D-lactic acidosis, and administration of chloride-rich solutions.^1^ After cross-referencing this with the case details, some of these diagnoses can be eliminated based on the history, exam, and review of systems. Specifically, the patient reported constipation, thereby eliminating diarrhea as a cause. She also had no surgical history, hence eliminating ureteroileostomy and ureterosigmoidostomy as causes. Although her diet is not mentioned, there is no reported history of abnormal ingestion of food or fluids; so I reasonably eliminated chloride-rich solution ingestion as a cause. This left proximal and distal RTA, toluene use, ketoacidosis, and D-lactic acidosis as diagnoses under consideration. When cross-referencing these with the case details and laboratory findings once again, some options were not consistent with the presentation. Specifically, there was no ketonuria making ketoacidosis unlikely. Lactic acidosis is a result of a hypoperfusion state, and the clinical case did not provide any evidence of hypoperfusion making this unlikely as well.

There were some additional laboratory findings outside of the metabolic panel that needed to be considered. Namely, the patient’s hemoglobin and hematocrit were slightly abnormal (although this may be due to her known anemia). Also, she had an elevated creatine kinase and myoglobin as well as slight elevation in her aspartate transaminase. Her urine also showed some hematuria, pyuria, and proteinuria as well as findings of nitrites and leukocyte esterase. When these labs are considered in conjunction with the metabolic abnormalities, my differential diagnosis now included hypokalemic periodic paralysis, rhabdomyolysis, adrenal insufficiency, proximal and distal RTA, inflammatory myopathy, and poisoning (including toluene).

Adrenal insufficiency can cause metabolic derangements and presents with symptoms including fatigue, weight loss, GI complaints, and myalgias, and may also include psychiatric symptoms. In primary adrenal insufficiency, the potassium is high and sodium is low, which is not consistent with this case. In secondary or tertiary adrenal insufficiency, potassium is normal or low, sodium can be high or low, and chloride is normal with a low glucose.^2^ These are not consistent with the findings in this case either; so I eliminated adrenal insufficiency from my differential diagnosis.

Inflammatory myopathies present with muscle weakness, cardiac involvement, and laboratory findings including elevated serum creatinine kinase and elevated myoglobin levels in both urine and serum. These patients usually present with acute onset of “antisynthetase syndrome,” constitutional symptoms, Raynaud’s phenomenon, and a nonerosive arthritis.^3^ While the laboratory findings here were consistent with a possible myopathy, the clinical presentation was not classic, making this a less likely possibility.

Another consideration was rhabdomyolysis potentially resulting from compartment syndrome. Compartment syndrome occurs from increased fascial compartment pressure with subsequent tissue hypoperfusion, which can lead to muscle necrosis and rhabdomyolysis. The classic triad of findings in rhabdomyolysis is muscle pain, weakness, and dark urine. Patients with rhabdomyolysis usually have some combination of highly elevated creatine kinase, myoglobinuria, hyperkalemia, hyperphosphatemia, acute kidney injury, hypocalcemia, and metabolic acidosis with or without an anion gap.^4^ In this patient’s case, there was no clear inciting event, and her symptoms were episodic with spontaneous resolution. Additionally, she did not complain of focal pain or weakness as would be expected in compartment syndrome. Although she did have an elevated creatine kinase, the elevation was not significant and the expected laboratory findings of hyperkalemia and hyperphosphatemia were not present. I felt that compartment syndrome and rhabdomyolysis were unlikely.

In this young adult patient with episodic weakness and hypokalemia, hypokalemic periodic paralysis was immediately considered as part of the differential diagnosis. This condition is characterized by attacks of weakness with a normal neurologic exam in between, as seen in this patient. Primary hypokalemic periodic paralysis follows an autosomal dominant inheritance pattern, and notably this patient had no known family history of the same. Bulbar and respiratory functions are preserved and between attacks, patients will also present with normal plasma potassium. Triggers include stress, exercise, and carbohydrates. The condition also presents with arrhythmias.^5^ There are, however, other conditions that can cause non-familial hypokalemic paralysis, including RTA.^6^

All three subtypes of RTA are characterized by an inability to acidify the urine. As a result of this, RTAs present with an increased urine anion gap, but this information was not provided in the case history. In distal or type 1 RTA, there is impaired hydrogen ion secretion in the distal tubule of the nephron. In proximal or type 2 RTA, there is impaired bicarbonate reabsorption in the proximal tubule of the nephron. In type 4 RTA, there is decreased aldosterone secretion or aldosterone resistance.^7^ As a result of this, type 4 RTA is associated with serum hyperkalemia while the other two types of RTA result in hypokalemia.^8^ Due to the serum potassium levels, which were not suggestive of aldosterone resistance, I eliminated type 4 RTA from my list of possibilities.

The types of hypokalemic RTAs are differentiated by examining the potential of hydrogen (pH) of the patient’s urine. In type 2 (proximal) RTA, urine pH is initially high, then decreases to < 5.5. The urine pH remains above 5.5 in type 1 (distal) RTA.^8^ This patient had a urine pH of 7.0, suggesting either a type 1 (distal) RTA or an early type 2 (proximal) RTA. Type 1 (distal) RTA can be hereditary or be caused by autoimmune diseases such as Sjögren’s syndrome, or as a complication of chemotherapy or toluene use. The causes of type 2 (proximal) RTA include genetic abnormalities, Fanconi syndrome, monoclonal gammopathy, and carbonic anhydrase inhibitor use.^7^ There was no mention of chemotherapy or carbonic anhydrase inhibitors with the patient’s presentation. The patient had no family history of similar issues, and it would stand to reason that a genetic abnormality would have come to light before age 19 years. As such, I feel type 1 (distal) RTA is more likely than type 2 (proximal) RTA.

The Agency for Toxic Substances and Disease Registry notes that toluene is a solvent found in paints, nail polish, paint thinners, and adhesives, among other substances. It can have toxic effects if ingested or inhaled.^9^ The findings of acute toluene use include a hypokalemic paralysis and a metabolic acidosis. Patients are also often found to have liver injury and rhabdomyolysis, and may present with altered mentation, renal failure, and acidemia.^10^

This patient’s presentation is most consistent with type 1 (distal) RTA due to toluene use. She denied any illicit drug use but did admit to a history of alcohol ingestion and marijuana use, raising the question of whether there could be toxic alcohols or other coingestions. Unfortunately, there is no diagnostic test for toluene use. However, proximal and distal RTA can be differentiated by calculating the urinary ammonium ion concentration from the measured urine anion gap and osmolar gap. Therefore, my test of choice would be a urine electrolyte panel to calculate the anion gap and osmolar gap. Additionally, I would consult nephrology to assist in management of this patient.

## CASE OUTCOME (DR. CAVALIERE)

The patient developed bigeminy while in the ED but remained hemodynamically stable and did not have a change in her mental status. Her electrolytes were replaced with oral and intravenous potassium, with improvement of her arrythmia and symptoms. She declined central line placement for more rapid replacement. The patient was admitted to the pediatric intensive care unit (PICU) for further management and evaluation.

Random urine electrolytes were obtained after the patient was admitted to the PICU (Table 2). Nephrology was consulted because these results demonstrated an elevated urine anion gap suggestive of RTA.

The patient’s symptoms completely resolved once her electrolytes were repleted. She was found to have positive antinuclear (ANA-Abs) and anti–Sjögren’s-syndrome-related antigen A (SS-A/Ro) autoantibodies, leading to the diagnosis of Sjögren’s syndrome, despite a lack of phenotypic features.

The patient’s urine anion gap was indeterminate for the etiology of her non-anion gap metabolic acidosis; however, her urine osmolar gap of less than 150 milliosmoles per kilogram (mOsm/kg) suggested type 1 or type 4 RTA as the etiology. This coupled with laboratory findings suggestive of autoimmune disease led to the diagnosis of type 1 RTA. Her RTA was treated with potassium supplementation and alkali therapy to achieve a normal serum bicarbonate concentration. Unfortunately, the patient has not been compliant with her home therapy and has required multiple hospitalizations since her original presentation. Her presentation and urine anion gap strongly suggest toluene toxicity, but the patient repeatedly denied insufflating glue and there is no diagnostic test for toluene.

## RESIDENT DISCUSION (DR. CAVALIERE)

Hyperchloremic, hypokalemic metabolic acidosis is a condition all emergency providers should be prepared to diagnose and manage. In this case, the patient presented with a cardiac arrhythmia due to severe hypokalemia. The underlying etiology of the hypokalemia should be sought while simultaneously treating the condition. The initial ED evaluation includes obtaining a basic metabolic panel and a urinalysis. Once it is determined that the patient does not have a serum anion gap, the clinician should consider three broad categories of non-anion gap acidosis and their etiologies: increased acid production; loss of bicarbonate; and decreased renal excretion of acid (Table 3).^11^

Type 1 or distal RTA is a primary problem of urine acidification due to impaired hydrogen ion secretion in the distal convoluted tubules. The underlying etiology in adults is usually autoimmune diseases such as Sjögren’s syndrome or rheumatoid arthritis.^12^ In pediatrics, the cause is usually a hereditary gene mutation for either the basolateral chloride-bicarbonate exchanger (SLC4A1) or the apical hydrogen-adenosine triphosphatase (ATP6V0A4 and ATP6V1B1) gene.^13^,^14^ Lastly, a distal RTA can be iatrogenic due to ifosfamide, a chemotherapeutic analog of cyclophosphamide.^15^

Type 2 or proximal RTA is a primary problem of impaired bicarbonate reabsorption leading to increased bicarbonate loss.^1^,^7^ In adults, the underlying etiology is most commonly proximal tubular toxicity from increased exertion of monoclonal immunoglobulin light chains as seen in multiple myeloma.^16^ Type 2 RTAs are seen in Fanconi syndrome (loss of bicarbonate with impaired reabsorption of phosphate, glucose, uric acid and amino acids), and in patients prescribed carbonic anhydrase inhibitors (acetazolamide or topiramate).^17^ In pediatric patients, type 2 RTAs are usually idiopathic, but they can be due to a complication from chemotherapy, cystinosis (genetic disorder causing an accumulation of cystine leading to crystal formation), or inherited mutations in the KCNJ15 (autosomal dominant) and SLC4A4 (autosomal recessive) genes.^16^,^18^,^19^ The term “type 3 RTA” is rarely used as it is now considered a combination of types 1 and 2. Type 4 (hyperkalemic) RTA is beyond the scope of this discussion.

The test of choice when evaluating for a RTA is urine electrolytes so that the clinician can calculate how much ammonium is being excreted.^18^ Ammonium excretion will be decreased in a true RTA and normal/increased if the acidosis is due to toluene use or chronic diarrhea. Unfortunately, ammonium excretion is rarely measured directly. Urine ammonium excretion can be estimated by using the urine anion gap or urine osmolar gap.^20^ The urine anion gap is calculated using the equation:

$$\text{Urine anion gap}={\left[\text{Sodium}\right]}_{\text{urine}}+{\left[\text{Potassium}\right]}_{\text{urine}}-{\left[\text{Chloride}\right]}_{\text{urine}}$$

A urine anion gap of 20–90 millimoles per liter (mmol/L) signifies impaired renal ammonium excretion and is diagnostic of a distal RTA. A value of −20 to −50 mmol/L is diagnostic of diarrhea and ingestions, as it indicates increased ammonium excretion. Values between −20 and 20 mmol/L are considered indeterminate.

Urine osmolar gap, on the other hand, is more difficult to calculate. The urine osmolar gap is the difference between the measured and the calculated urine osmolarity. Urine osmolarity is calculated using the equation:

$$\text{Urine osmolarity }(\text{milliosmoles}/\text{kg})=2({\left[\text{Sodium}\right]}_{\text{urine}}+{\left[\text{Potassium}\right]}_{\text{urine}})+{\left[\text{Urea}\right]}_{\text{urine}}+{\left[\text{Glucose}\right]}_{\text{urine}}$$

Values less than 150 mOsm/kg are consistent with a type 1 or 4 RTA, while values greater than 400 mOsm/kg are consistent with either diarrhea or toluene use.^20^

Patients with acute presentations of type 1 or 2 RTA should have their electrolytes replaced emergently to prevent cardiac arrythmias. Central line insertion and high concentration potassium replacement should be considered in severe, symptomatic hypokalemia. Chronic type 1 RTA is treated with alkali therapy to achieve a normal serum bicarbonate concentration (22–24 mmol/L).^21^ The long-term treatment of type 2 RTA depends upon the underlying etiology.^22^

## FINAL DIAGNOSIS

Type 1 (distal) renal tubular acidosis with positive SS-A/Ro antibodies.

## KEY TEACHING POINTS

- Type 1 (distal) RTA is an uncommon cause of a non-anion gap metabolic acidosis.
- A type 1 RTA can be diagnosed using a basic metabolic panel, the urine pH from a urinalysis, and by measuring urine electrolytes.
- An elevated urine anion gap or decreased urine osmolar gap will clinch the diagnosis of a type 1 RTA.

## Figures and Tables

**Image:**
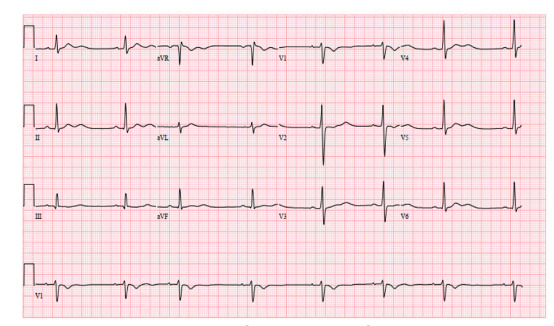
Electrocardiogram of a 19-year-old female with weakness.

**Table 1:** Initial laboratory results of a 19-year-old female with weakness.

Laboratory test	Patient value	Normal value
Complete blood count		
White blood cells	7.7 K/mcL	3.4 – 10.8 K/mcL
Hematocrit	31.9%	34.0 – 46.6%
Platelets	239 K/mcL	150 – 450 K/mcL
Serum chemistries		
Sodium	139 mmol/L	134 – 144 mmol/L
Potassium	1.3 mmol/L	3.5 – 5.2 mmol/L
Chloride	116 mmol/L	96 – 106 mmol/L
Carbon dioxide	13 mmol/L	20 – 29 mmol/L
Blood urea nitrogen	17 mg/dL	5 – 18 mg/dL
Creatinine	1.16 mg/dL	0.49 – 0.90 mg/dL
Glucose	88 mg/dL	65 – 99 mg/dL
Calcium	10.4 mg/dL	8.9 – 10.4 mg/dL
Magnesium	2.7 mg/dL	1.6 – 2.6 mg/dL
Phosphorus	2.3 mg/dL	2.5 – 4.5 mmol/dL
Total protein	2.3 g/dL	6.3 – 8.2 g/dL
Albumin	4.6 g/dL	3.7 – 5.6 g/dL
Aspartate aminotransferase	65 u/L	14 – 36 u/L
Alanine aminotransferase	31 u/L	0 – 34 u/L
Total bilirubin	0.3 mg/dL	0.2 – 1.3 mg/dL
Alkaline phosphatase	115 u/L	38 – 126 u/L
Additional labs		
Creatinine kinase	1117 u/L	30 – 135 u/L
Myoglobin	599 ng/mL	≤ 62 ng/mL
Thyroid stimulating hormone	0.850 mcIU/L	0.450 – 5.330 mcIU/mL
Human chorionic gonadotropin	Negative	Negative
Urine toxicology screen	Cannabis present	Negative
Urinalysis		
Color	Straw	
Appearance	Slightly cloudy	Clear
Specific gravity	1.004	1.002 – 1.030
pH	7.0	5.0 – 8.0
Glucose	Negative	Negative
Bilirubin	Negative	Negative
Urobilinogen	Negative	Negative
Ketones	Negative	Negative
Blood	2+	Negative
Protein	1+	Negative
Nitrite	Positive	Negative
Leukocyte esterase	1+	Negative
White blood cells	11–25 cells/hpf	0 – 5 cells/hpf
Red blood cells	0–2 cells/hpf	0 – 2 cells/hpf
Squamous epithelial cells	0–2 cells/hpf	0 – 2 cells/hpf
Bacteria	Small	Negative

*cells/hpf*, cells per high powered field; *g/dL*, grams per deciliter; *K/mcL*, thousand cells per microliter; *mcIU/mL*, micro-international units per milliliter; *mg/dL*, milligram per deciliter; *mmol/L*, millimole per liter; *ng/mL*, nanogram per milliliter; *u/L*, units per liter.

**Table 2:** Urine electrolytes of a 19-year-old female with weakness.

Urine electrolyte	Patient value	Normal value
Chloride	25 mmol/L	32 – 290 mmol/L
Creatinine	24.6 mg/dL	20–275 mg/dL
Potassium	11.5 mmol/L	12–129 mmol/L
Sodium	31 mmol/L	28 – 272 mmol/L
Urea	109 mg/dL	---
Glucose	0 mg/dL	0 mg/dL
Osmolality	259 mOsm/kg	50 – 1200 mOsm/kg
Urine anion gap	17.5 mmol/L	0 mmol/L
Urine osmolar gap	146.57 mOsm/kg	80–100 mOsm/kg

*mg/dL*, milligram per deciliter; *mmol/L*, millimole per liter; *mOsm/kg*, milliosmoles per kilogram.

**Table 3:** Categories and etiologies of non-anion gap metabolic acidosis^1^,^7^,^11^,^12^

Category	Etiology
Increased acid production	Lactic acidosis
	Ketoacidosis (starvation, alcoholic and diabetes ketoacidosis)
	Ingestions (methanol, ethylene glycol, aspirin, toluene, diethylene glycol, propylene glycol, D-lactic acidosis)
Loss of bicarbonate	Diarrhea
	Type 2 (proximal) RTA
	Ketoacidosis recovery
	Carbonic anhydrase inhibitor use
	Ureteral diversion (ureteroileostomy, ureterosigmoidostomy)
Decreased renal excretion of acid	Type 1 (distal) RTA, Type 4 RTA

*RTA*, renal tubular acidosis.
